# Controllable self-assembly of thiophene-based π-conjugated molecule and further construction of pillar[5]arene-based host-guest white-light emission system

**DOI:** 10.3389/fchem.2022.980173

**Published:** 2022-09-02

**Authors:** Haibo Zhong, Liang Li, Shajun Zhu, Yang Wang

**Affiliations:** ^1^ School of Chemical and Environmental Engineering, Shanghai Institute of Technology, Shanghai, China; ^2^ School of Chemistry and Chemical Engineering, Nantong University, Nantong, China; ^3^ Department of Hepatobiliary and Pancreatic Surgery, Affiliated Hospital of Nantong University, Nantong, China

**Keywords:** self-assembly, pillar[5]arene, thiophene, π-Conjugated molecule, white-light emission

## Abstract

Photoluminescence materials have been widely applied in biological imaging and sensing, anti-counterfeiting, light-emitting diodes, logic gates et al. The fabrication of luminescent materials with adjustable emission color by self-assembly of π-conjugated molecules has attracted particular attention. In this study, we designed and synthesized a thiophene-based α-cyanostyrene-derivative (TPPA), then investigate its self-assembly morphology and fluorescence emission under different organic solvents, different proportions of H_2_O/THF (DMSO) mixture and different pH conditions by UV, FL and SEM images. It was found that TPPA formed nanoparticles by self-assembly in organic solvent (THF or DMSO), accompanied by strong fluorescence emission. However, with the increase of water ratio, the fluorescence intensity decreased accompany with red shift, and the self-assembly morphology changed from nanoparticles to fibers. More interestingly, when pillar[5]arene (P5) was added to form host-guest complex with TPPA, white light emission could be successfully constructed when the ratio of TPPA to P5 was 1:20 and THF to water was 19:1.

## Introduction

In recent years, highly efficient photoluminescent materials with tunable multicolor luminescence properties have been widely applied in biological imaging and sensing (Yang et al., 2013; Guo et al., 2020a; Zhao et al., 2021; Wang et al., 2022a), anti-counterfeiting (Yu et al., 2020; Yang et al., 2021), light-emitting diodes (Fung et al., 2016), molecular switches and logic gates (Erbas-Cakmak et al., 2018). At present, the main strategy for the construction of luminescent materials is physical mixing or covalently linking complementary chromophores to achieve appropriate color mixing balance (Park et al., 2009; Peng et al., 2021). Among all kinds of luminescent materials, white luminescent materials are particularly valued because they are key components in various display and lighting applications (Aizawa et al., 2014; Sun et al., 2018). Compared with physical mixing and covalent bonding, the luminescent materials based on self-assembly have the advantages of modularization, simple synthesis and adjustable properties, so they have a broad application prospect (Wang et al., 2019; Li L. et al., 2020; Zhang Y. et al., 2020; Cai et al., 2020; Liu M. et al., 2021). By applying a variety of external stimuli, such as solvent polarity, light exposure, mechanical/thermal stimulation and humidity, the emitted colors can be effectively regulated, which also provides an environmentally friendly method for organic modules to prepare luminescent materials in aqueous media (Zhu et al., 2013; Wei et al., 2016; Cheng et al., 2017; Fang et al., 2017). Since the modulation of π-conjugated dyes in terms of their optical properties depends largely on the way the molecules are arranged, the effective manipulation of their emission can be achieved by adjusting the morphology of the photoluminescent proto-components, which is also critical in preparing valuable materials (Ostroverkhova 2016; Kundu et al., 2021; Lu et al., 2021). Thiophene-based α-cyanostilbene derivatives are a typical class of π-conjugated molecules (Yun et al., 2012; Martínez-Abadía et al., 2018). In addition to their interesting electrical properties, these compounds also have significant optical properties, so they are considered to be a very suitable and general choice for the development of functional materials (Bhaumik and Banerjee., 2020; Wang X.-H. et al., 2021; Li et al., 2021).

Pillar[*n*]arenes (Ogoshi et al., 2008; Xiao et al., 2018; Duan et al., 2020; Wang et al., 2020; Wang et al., 2022b) are the fifth generation of macrocyclic hosts following crown ethers (An et al., 2021), cyclodextrins (Zhou et al., 2021), calixarenes (An et al., 2019; Guo et al., 2020b) and cucurbiturils (Yan et al., 2021). They are oligo-cyclic compounds obtained by methylene bridged *p*-methoxylbenzene (Zhang et al., 2019; Lou and Yang, 2021). Various functional groups can be easily modified to pillar[*n*]arenes by reacting with phenolic hydroxyl groups along the upper and lower edges of the pillar[*n*]arene-frameworks (Zhang R. et al., 2020; Guo et al., 2021; Schmidt and Esser, 2021). In addition, the adjustable cavity size of pillar[*n*]arenes also endow them with rich host-guest properties (Wang M. et al., 2021; Cai et al., 2021; Wang Y. et al., 2022), such as alkyl chain guests trend to complex with pillar[5]arene, while pyridinium guests trend to complex with pillar[6]arene (Guo et al., 2020c; Shen et al., 2020; Liu D. et al., 2021). In recent years, pillar[*n*]arenes have developed rapidly from synthesis (Ma et al., 2019), host-guest interaction (Li B. et al., 2020; Huang et al., 2020) to functional derivation (Wu et al., 2018), and have been successfully applied to gas separation, ion detection, drug release, tumor therapy, optical materials et al. (Cen et al., 2020; Liu X. et al., 2021; Xiao et al., 2022). In particular, the pillar[*n*]arene-based photoluminescent materials are of particular interest because of their multiple stimulus responsiveness and controllable optical properties (Sun et al., 2021). For example, Yang and *co*-workers fabricated a non-metallic white light-emitting fluorescent material based on pillar[5]arene-tripoxamide system, and found that supramolecular assembly plays a key role in the process of white light-emitting (Yang et al., 2020).

**SCHEME 1:**
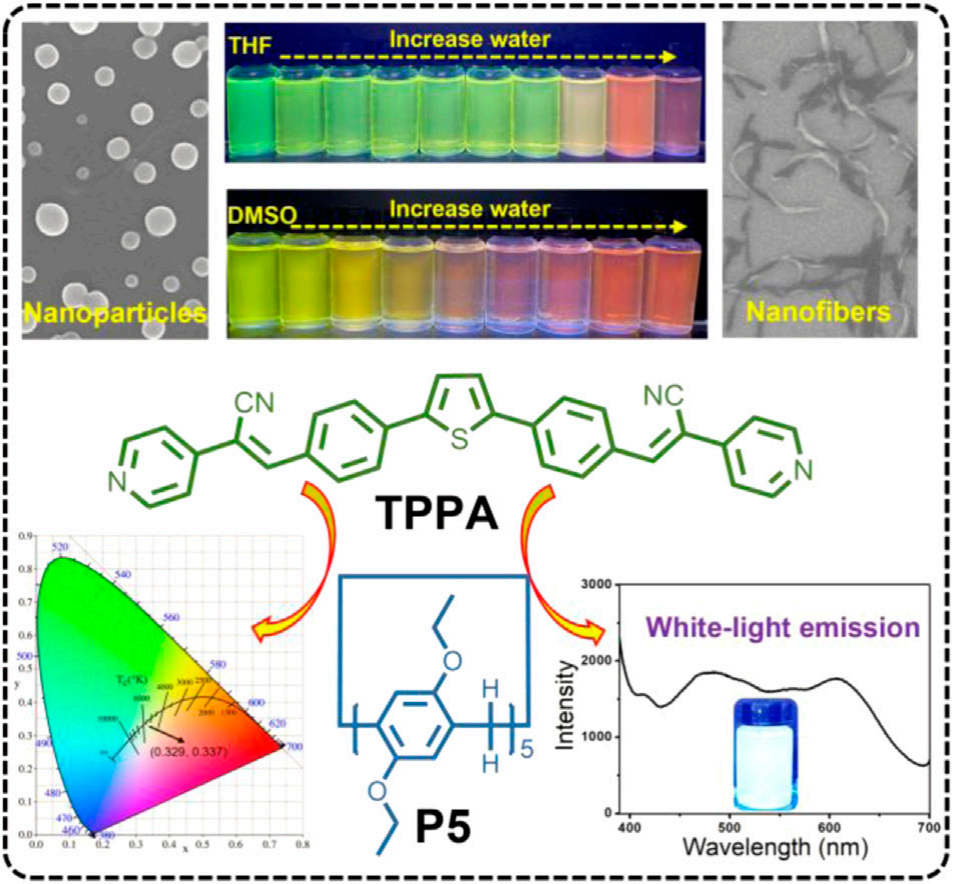
Chemical Structures of thiophene-based α-cyanostyrene-derivative (TPPA), and pillar[5]arene (P5) and Cartoon Representation of TPPA self-assembly in various solvents and further construction of pillar`[5]arene-based host-guest white-light emission system.

In this study, we constructed a new white light emission material based on the *co*-assembly of pillar[5]arene and thiophene-based α-cyanostyrene-derivative (TPPA). TPPA was observed to form nanoparticles when self-assembled in organic solvent (THF or DMSO), accompanied with bright fluorescence emission. However, with the increase of water ratio, the fluorescence intensity decreased sharply accompany with red shift, and the morphology of the assemblies changed from nanoparticles to nanofibers (Scheme 1). Importantly, when pillar[5]arene (P5) was added to form host-guest complex with TPPA, white light emission could be successfully constructed in THF/H_2_O mixture (Scheme 1).

## Experiment section

### Synthesis of thiophene-based α-cyanostyrene-derivative (TPPA)

**SCHEME 2:**
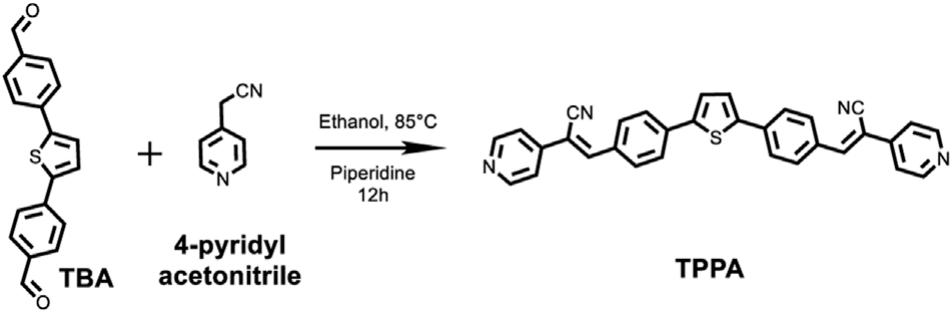
Synthetic route to (2Z,2′Z)-3,3′-(thiophene-2,5-diylbis (4,1-phenylene))-bis(2-(pyridin-4-yl)acrylonitrile) (TPPA).

TBA (0.34 mmol), 4-pyridylacetonitrile (0.68 mmol) and piperidine (1.36 mmol) were dissolved in CH_3_CH_2_OH and stirred inside a Schlenk tube. The reaction mixture was heated to 85°C overnight under continuous stirring. Then, the obtained orange precipitate was washed several times with ethanol and hexane through centrifugation. An orange-colored solid was obtained with a 48% yield (Scheme 2).

**SCHEME 3:**
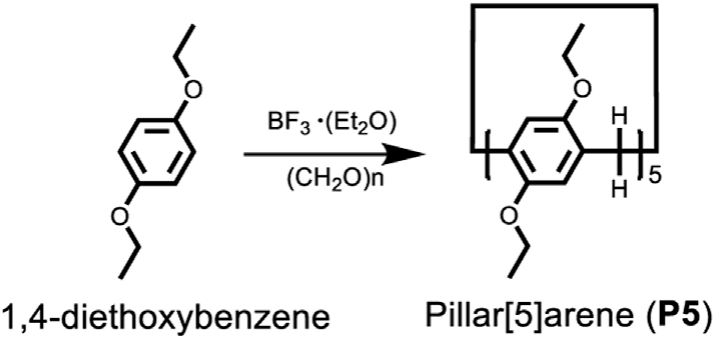
Synthetic route to pillar[5]arene (P5).

TPPA: orange-colored solid, yield 48%. ^1^H NMR (Supplementary Figure S1) (400 MHz, Chloroform-d) δ 8.73–8.70 (m, 4H), 8.01 (d, J = 8.2 Hz, 4H), 7.78 (d, *J* = 8.1 Hz, 4H), 7.73 (s, 2H), 7.60 (d, 4H), 7.49 (s, 2H). ^13^C NMR (Supplementary Figure S2) (101 MHz, CDCl_3_) δ 150.54, 144.19, 143.66, 141.95, 136.97, 130.66, 126.00, 119.98, 117.06, 108.65. MS (ESI) (Supplementary Figure S3) Calcd. for C_32_H_20_N_4_SNa ([M + Na]^+^): 515.1, found: 515.1.

### Synthesis of pillar[5]arene (P5)

Pillar[5]arene was prepared according previous report, in a typical process (Scheme 3), 1,4-diethoxybenzene (1.66 g, 1.0 mmol) and paraformaldehyde (0.30 g) were added to 60 ml ClCH_2_CH_2_Cl under vigorous stirring at room temperature. 1 ml BF_3_(Et_2_O) was added to the mixture and then reacted for 1 h. 50 ml H_2_O was added to stop the reaction, and pillar[5]arene was obtained by column chromatography (volume ratio: dichloromethane: petroleum ether = 1 : 1). White solid, 80%; ^1^H NMR (Supplementary Figure S4) (400 MHz, CDCl_3_) δ 6.72 (s, 10H, ArH), 3.83 (20H, OCH_2_-), 3.76 (s, 10H, ph-CH_2_-ph), 1.26 (t, *J* = 6.00 Hz, 30H, CH_3_).

## Materials and methods

The TBA was prepared according to previous report. 4-pyridylacetonitrile, 1,4-diethoxybenzene and the reagents (ethanol, piperidine, ClCH_2_CH_2_Cl and so on) were commercially available (99%) and used as received. Further purification and drying of the solvents by standard methods were employed and distilled prior to use when necessary.

^1^H NMR and ^13^C NMR spectra were recorded on a Bruker AVIII-400 MHz spectrometer. All NMR used tetramethylsilane (TMS) as the internal standard. Bruker Micro-TOF spectrometer was used to investigate the High-resolution Mass (ESI) of the compounds. Fluorescence spectra were recorded on a Hitachi F-7000FluorescenceSpectrophotometer. Confocal images were acquired using an Olympus FLUOVIEWFV1000confocallaser scanning unit mounted on an IX81 fixed stage upright microscope. Scanning electron microscopy (SEM) investigations were carried out on a JEOL6390LVinstrument.

## Results and disscussion

### Impact of solvents

**FIGURE 1:**
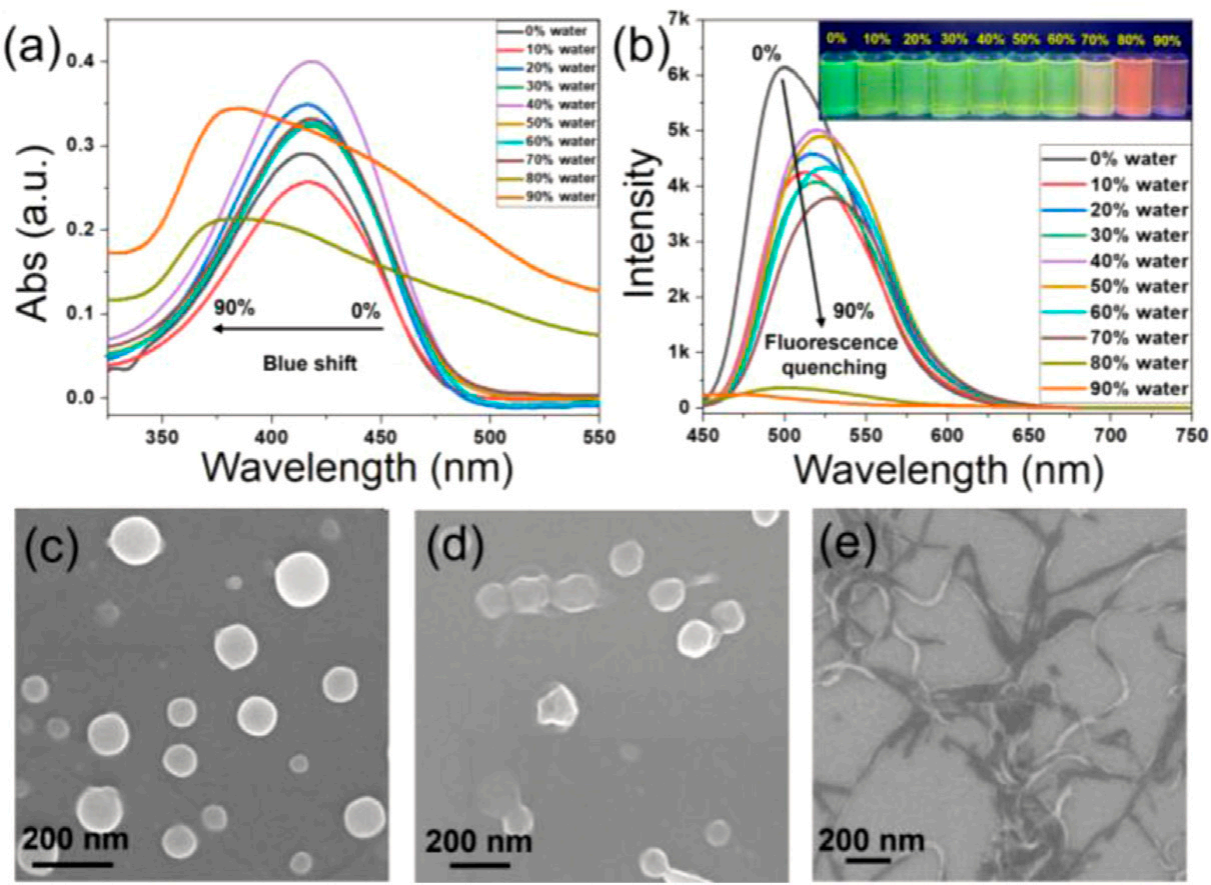
**(A)** absorption and **(B)** emission spectra of TPPA (0.4 mM, 4 ml) in 10 ml THF/water mixture with varying the water fraction from 0 to 90% with 10% of interval. **(C)** 0%, **(D)** 70%, and **(E)** 90% water content illustrating aggregation of spherical nanoparticles (0% W), semi-spherical oblong-shaped nanoparticles (70% W) to 1D-nanofibers (90% W).

The obtained TPPA can be dissolved in most organic solvents and the corresponding solutions are stable as no precipitations were observed overnight. As shown in Supplementary Figure S5A, all the solution of TPPA displayed the characteristic band at about 420 nm with the similar intensity. Fluorescence emission spectra of TPPA showed a characteristic band at about 530 nm (Supplementary Figure S5B), and the intensity in large polarity solvent (CH_3_CN) is much lower than in smaller polarity solvent (Toluene). We further investigated the optical properties of TPPA in water/THF binary mixture, and the water content in the system gradually increased from 0% to 90%. A blue-shift was observed in UV-vis spectra with the increase of water content, and a dramatical change was found when the water content reached 80% (Figure 1A). On the other hand, a red-shift was found in fluorescence spectrum with the increase of water content. What’s more, the intensity of the peak decreased, and the fluorescence quantum yield decreased from 9.5% to 2.5% when the water increased from 0% to 90%, which indicated the formation of aggregates (Supplementary Table S1).

The morphology of the TPPA in THF/H_2_O mixture was observed through SEM. SEM images clearly show that with the water fraction increased from 0% to 70% and finally to 90%, the morphology of the assemblies gradually changes from nanoparticles to oblong-shaped nanoparticles and finally to nanofibers. This indicates that water fraction plays a decisive role in the transformation of aggregate morphology. We further studied the fluorescence changes of TPPA in DMSO/H_2_O mixture to reveal the nature of fluorescence. Both UV and fluorescence spectra showed abrupt spectral when the water fraction reaches 40% (Supplementary Figure S6). At the same time, the aggregation curve of TPPA in DMSO/H_2_O also confirmed that TPPA aggregated when the water content is between 30% and 40%, which is significantly lower than the water content when TPPA aggregated in H_2_O/THF mixture, because the polarity of DMSO is greater than THF, indicating that the polarity of solvent plays a key role in the assembly behavior of TPPA.

### Role of pH

**FIGURE 2:**
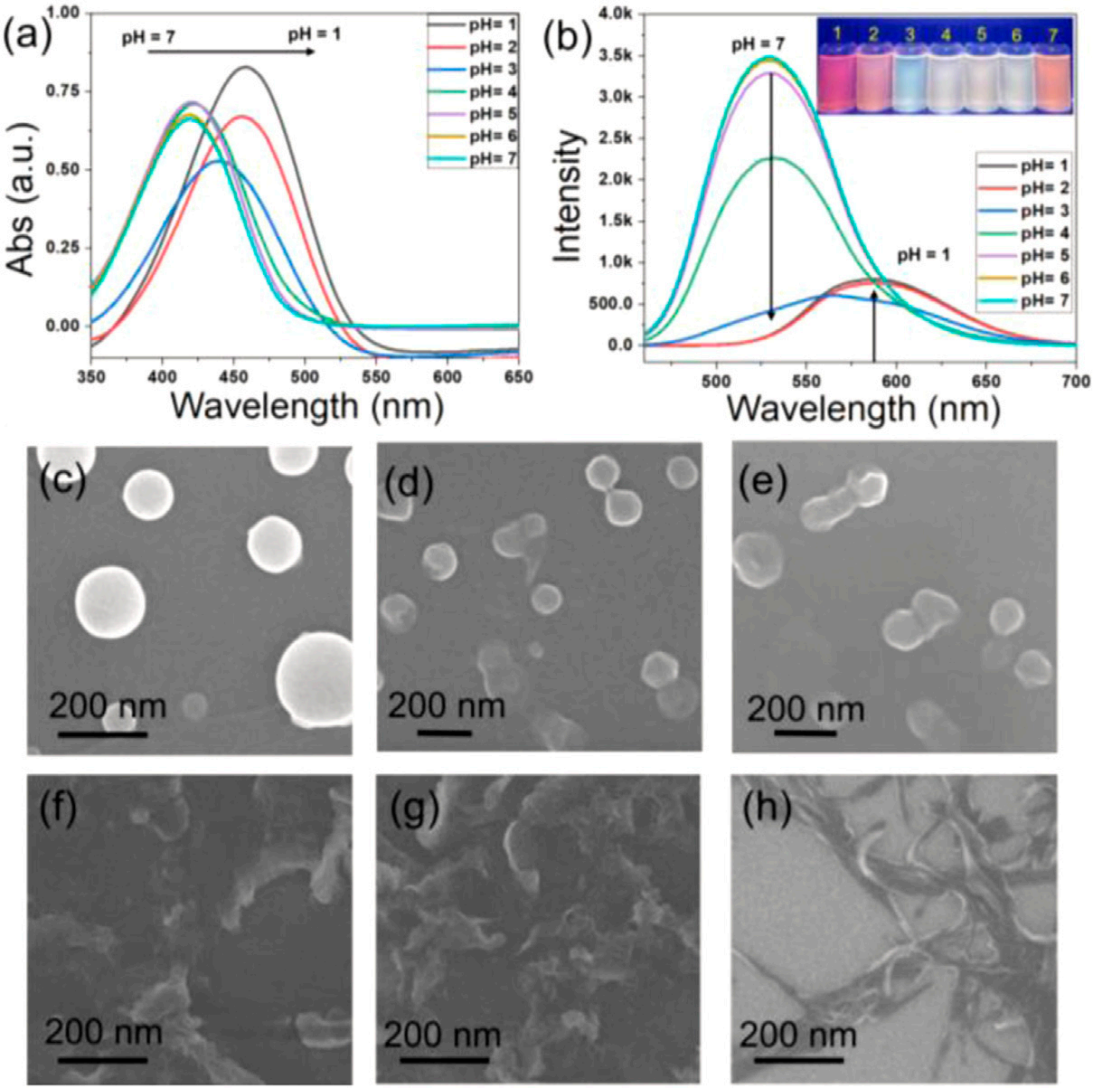
**(A)** absorption and **(B)** emission spectra of TPPA (0.4 mM, 4 ml) in 10 ml THF/water mixture (THF/H_2_O = 2:8) with different pH. SEM images of TPPA self-assembly in THF/water mixture (THF/H_2_O = 2:8) with different pH **(C)** pH = 1, **(D)** pH = 2, **(E)** pH = 3, **(F)** pH = 5, **(G)** pH = 6, **(H)** pH = 7.

The molecular structure, along with the polarity of the medium, plays a significant role in the formation of anisotropic nano-assembly. Because the pyridine N in TPPA can combine with H ion to change the polarity of the molecule, further affecting the optical properties and assembly behavior of the molecule in solution. The UV-vis spectra showed that the characteristic absorption peak have a significant red shift as pH decreased from 7 to 1, and the positions of the absorption peaks change dramatically when pH is between 3 and 4 (Figure 2A). Fluorescence spectra showed that the peak at 520 nm gradually decreased with the decrease of pH, but the peak at 600 nm gradually increased, indicating the formation of a new assembly morphology (Figure 2B). Furthermore, SEM images revealed a gradual disaggregation of the pristine nanofibers to nanoparticles with pH due to the protonation of the pyridinic nitrogen center upon the addition of acid (Figures 2C–H).

### Living cell imaging

**FIGURE 3:**
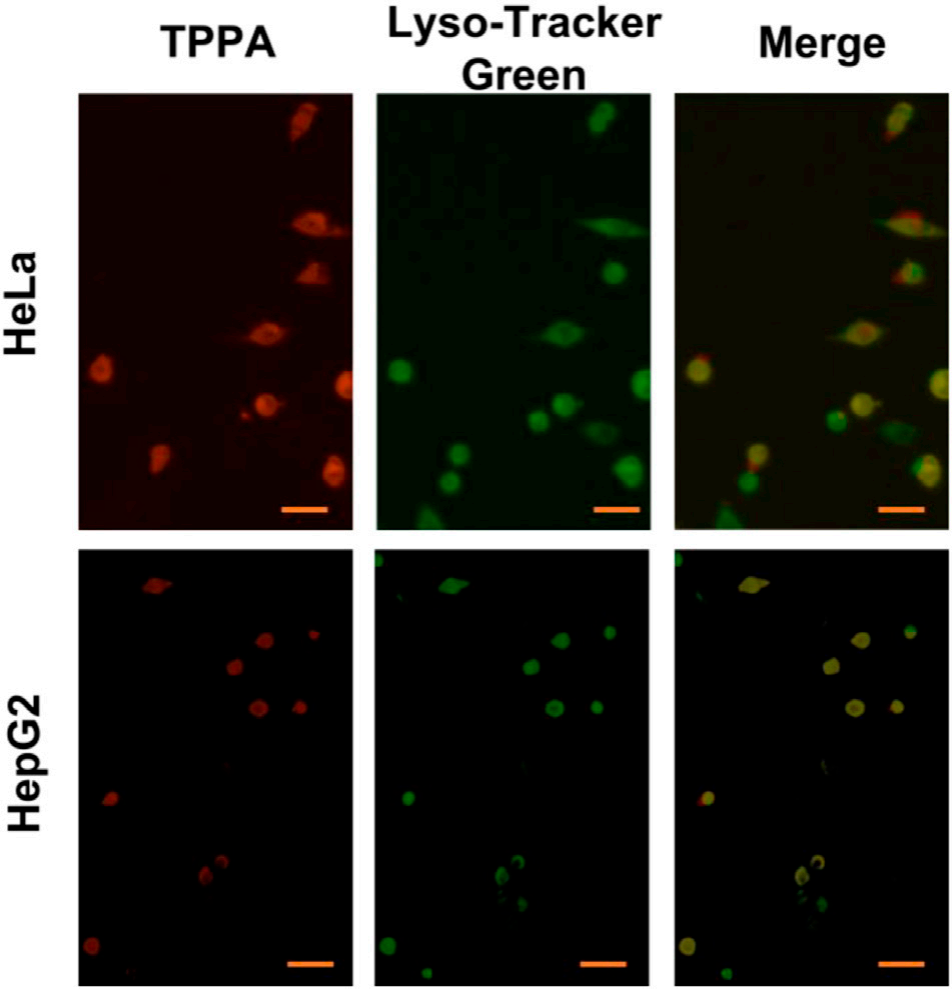
Confocal images of live HeLa and HepG2 cells after incubation with TPPA (5.00 × 10^−4^ M) for 4 h. Scale bar is 50 μm.

Due to TPPA can self-assembly into fluorescent nanostructures, we wondered whether they could be applied in the field of biomedical detection. At first, the toxicity of TPPA to Hela cells was evaluated by 3-(4,5-dimethylthiazol-2-yl)-2,5-diphenyltetrazolium bromide (MTT) assay. After co-culture of Hela cells with TPPA at concentrations ranging from 5.0 to 80 μg/ML for 4 h, the viability of HeLa cells was basically unchanged, indicating that the TPPA-based nanostructure has good cellular compatibility and very low cytotoxicity. Subsequently, we used TPPA as a cell imaging reagent. After HeLa and HepG2 cells were treated with TPPA for 4 h, the distribution of TPPA in Hela and HepG2 cells was monitored by confocal laser scanning microscopy (CLSM). As shown in Figure 3, both TPPA-treated HeLa and HepG2 cells showed bright red fluorescence in the lysosomal of the cells, indicating that TPPA can be successfully used for live cell imaging.

### White-light emission

**FIGURE 4:**
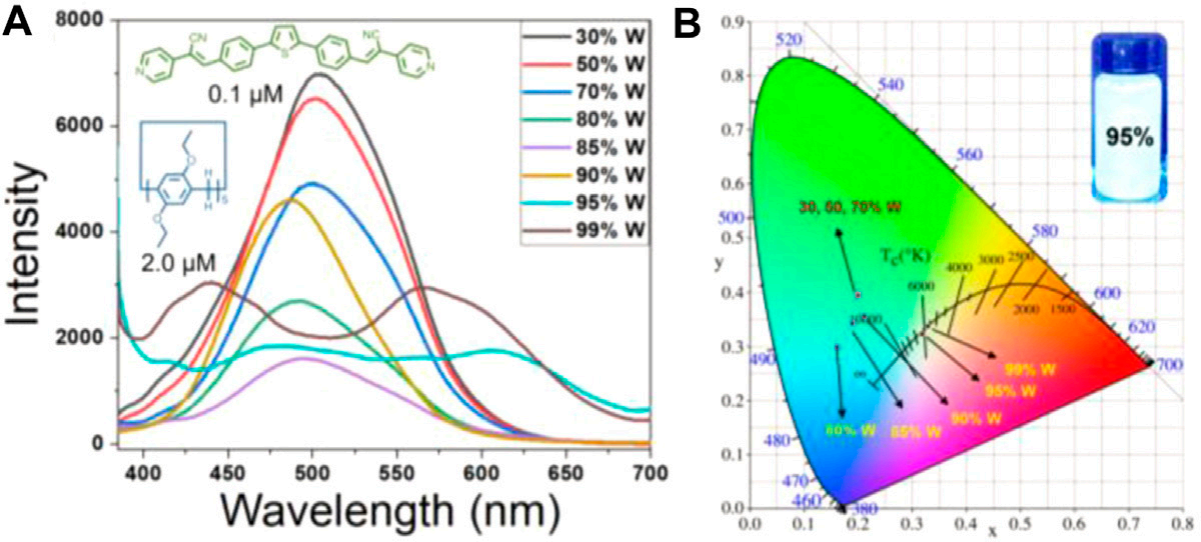
**(A)** Fluorescence spectra of 0.1 μmol TPPA and 2.0 μmol P5 in different ratio of THF/H_2_O mixture. **(B)** CIE chromaticity coordinates of TPPA&P5 according to the spectra recorded in **(A)**. inset: luminescence image of TPPA&P5 under 365 nm UV light.

Over the past decade, white light emitting materials have attracted much attention due to their potential applications in display technology and fluorescence sensors. In this work, a simple and efficient way for constructing white light-emitting material through the assembly between methoxyl pillar[5]arene (P5) and TPPA. From 2D NMR (Supplementary Figure S9) and IR (Supplementary Figure S10) spectra, we found P5 could provide C−H···π acting force and rich electronic cavity while the pyridine groups of the TPPA serve as electron-deficient sites. First, we fixed the amount of TPPA (0.1 μmol) in H_2_O/THF mixture and gradually increased P5. We find that with the increase of P5, the peak at 500 nm decreases and the peak at 650 nm increases, while when P5/TPPA is greater than 20, the peak at 650 nm decreases and the peak at 600 nm increases, indicating that the system may present white emission when P5/TPPA is around 20 (Supplementary Figure S8). We then fixed P5/TPPA at 20:1 and changed the ratio of THF to water in the mixture. As shown in Figure 4A, the peak intensity decreased with the increase of water content from 30% to 80%, while the peak intensity increased when the water content larger than 90%. As shown in Figure 4B, the system 0.1 μmol TPPA and 2.0 μmol P5 in THF/H_2_O mixture (5% THF and 95% water) was perceived as white light emitting with color coordinates of (0.329, 0.337), and the fluorescence quantum yield was 2.05 ± 0.06. The coordinate is very close to the pure white point (0.333, 0.333). In this case, the luminescence covers the entire visible spectral region (400–700 nm), giving our system overall white light emission (Figure 4B). It should be pointed that the morphology of TPPA was transformed from nanofibers to microparticles after addition of P5 (Supplementary Figure S11), and these particles can also be used for living cell imaging (Supplementary Figure S12).

## Conclusion

In this paper, a new π-conjugated molecule thiophene-based α-cyanostyrene-derivative (TPPA) was designed and synthesized successfully. TPPA showed bright fluorescence when dissolving in different organic solvents, and the fluorescence intensity increased with the decrease of the solvent polarity. Further investigation of TPPA in THF(DMSO)/H_2_O mixture found that TPPA formed nanoparticles by self-assembly in organic solvent (THF or DMSO), accompanied by strong fluorescence emission. However, with the increase of water ratio, the fluorescence intensity decreased accompany with red shift, and the self-assembly morphology changed from nanoparticles to fibers. Importantly, when macrocyclic host pillar[5]arene (P5) was added to form host-guest complex with TPPA, white light emission could be successfully constructed. This work provided a useful strategy for construction of photoluminescent materials based on supramolecular self-assembly.

## Data Availability

The original contributions presented in the study are included in the article/Supplementary Material, further inquiries can be directed to the corresponding authors.
