# Supplementary material for: Controllable self-assembly of thiophene-based π-conjugated molecule and further construction of pillar[5]arene-based host-guest white-light emission system
Source: Front Chem. 2022 Sep 2;10:980173. doi: 10.3389/fchem.2022.980173 (PMC9478560; doi:10.3389/fchem.2022.980173)
Supplement: Supplementary file 1 [file DataSheet1.docx]

Controllable self-assembly of thiophene-based π-conjugated molecule and further construction of pillar[5]arene-based host-guest white-light emission system

*Haibo Zhong^1,2^, Liang Li^1,*^ Shajun Zhu,^3*^ and Yang Wang^2,*^*

*^1^School of Chemical and Environmental Engineering, Shanghai Institute of Technology, Shanghai, 201418, P. R. China*

*^2^School of Chemistry and Chemical Engineering, Nantong University, Nantong, Jiangsu, 226019, P.R. China*

*^3^Department of Hepatobiliary and Pancreatic Surgery, Affiliated Hospital of Nantong University, Nantong, 226001, P.R. China*

**Supporting Information**

| 1 | Materials and methods | S2 |
| --- | --- | --- |
| 2 | Synthesis of **TPPA** | S3 |
| 3 | Synthesis of **P5** | S5 |
| 4 | Impact of solvents | S6 |
| 5 | Cell viability | S8 |
| 6 | White-light emission | S8 |

1. Materials and methods

**Materials**

All reagents were commercially available and used as supplied without further purification. Solvents were either employed as purchased or dried according to procedures described in the literature.

**Measurements**

**NMR spectroscopy.** ^1^H and ^13^C NMR spectra were recorded on a Brucker AV400 spectrometer.

**Fluorescence spectroscopy.** Steady-state fluorescence spectra were recorded in a conventional quartz cell (light path 10 mm) on a Varian Cary Eclipse equipped with a Varian Cary single-cell peltier accessory to control temperature.

**UV/Vis spectroscopy.** UV/Vis spectra and the optical transmittance were recorded in a quartz cell (light path 10 mm) on a Shimadzu UV-3600 spectrophotometer equipped with a PTC-348WI temperature controller.

**ESI-MS spectroscopy.** Electrospray ionization mass spectra (ESI-MS) were measured by Agilent 6520 Q-TOF-MS.

**Cytotoxicity experiments.** HeLa and HepG2 cells were incubated in Dulbecco’s modified Eagle’s medium (DMEM). The medium was supplemented with 10% fetal bovine serum and 1% Penicillin-Streptomycin. Cells were seeded in 96-well plates (5 × 10^4^ cell mL^–1^, 0.1 mL per well) for 4 h at 37^o^C in 5% CO_2_. Then the cells were incubated with different groups for 4 h. The relative cellular viability was determined by the MTT assay.

**Confocal laser scanning microscopy.** Cells were seeded in 6-well plates (5 × 10^4^ cell mL^–1^, 2 mL per well) for 24 h at 37^o^C in 5% CO_2_. The cells were incubated with the corresponding solution for 4 h. Then the medium was removed, and the cells were washed with phosphate buffer solution for three time. Finally, the cells were subjected to observation by a confocal laser scanning microscope.

1. Synthesis of **TPPA**

Scheme S1. Synthetic scheme for TPPA through Suzuki coupling reaction.

**TBA** (0.34 mmol)^S1^ and 4-pyridylacetonitrile (0.68 mmol) were mixed in ethanol and stirred inside a Schlenk tube. A catalytic amount of piperidine (1.36 mmol) was added into the reaction tube. The reaction mixture was refluxed at 85 °C for 12 h under continuous stirring. Then, the obtained orange precipitate was washed several times with ethanol and hexane through centrifugation. An orange-colored solid was obtained with a 48% yield.

^1^H NMR (Scheme S1) (400 MHz, Chloroform-*d*) δ 8.73 – 8.70 (m, 4H), 8.01 (d, *J* = 8.2 Hz, 4H), 7.78 (d, *J* = 8.1 Hz, 4H), 7.73 (s, 2H), 7.60 (d, 4H), 7.49 (s, 2H).


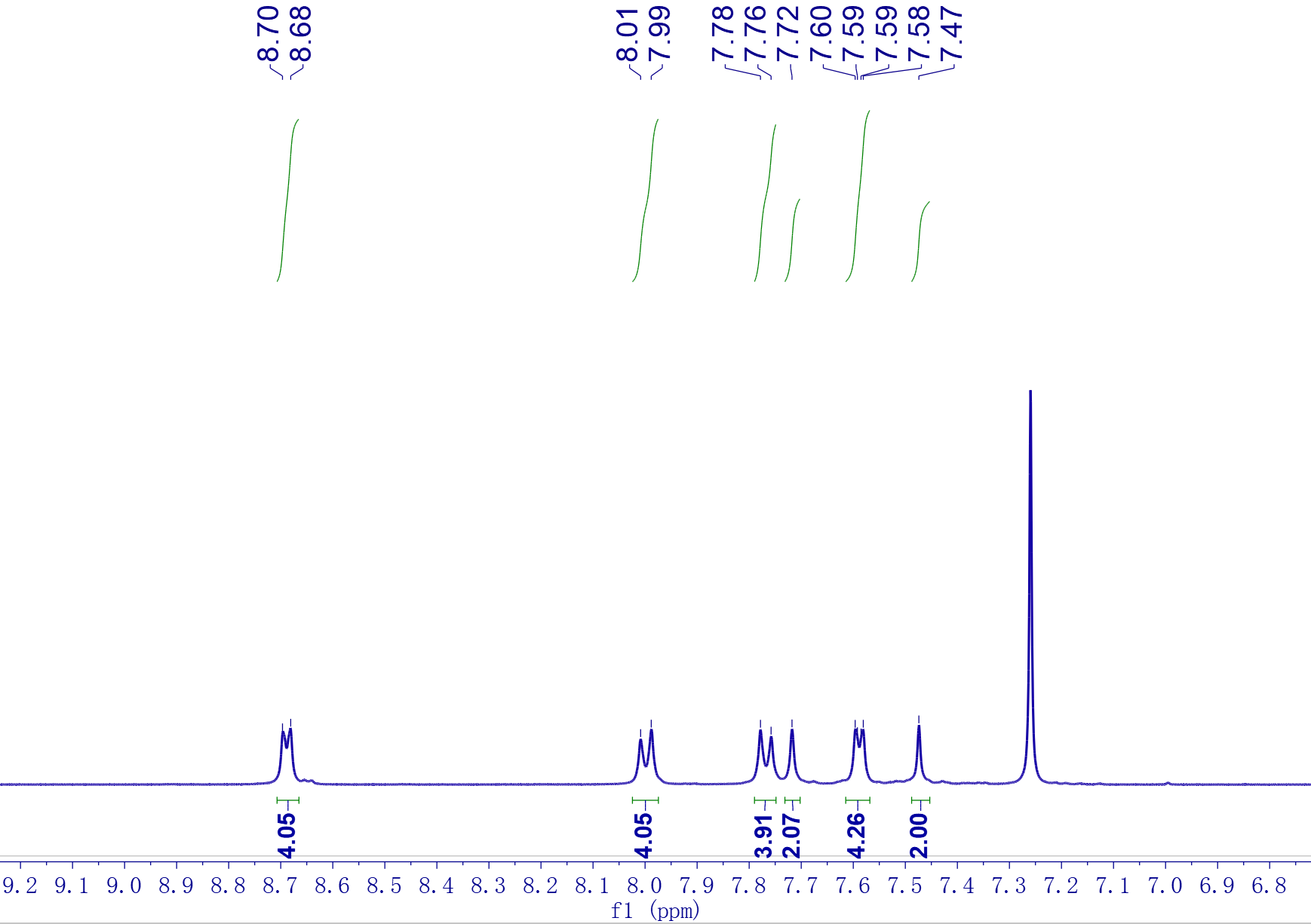


Figure S1. ^1^HNMR (400 MHz, 298K, CDCl_3_) spectrum of **TPPA**.


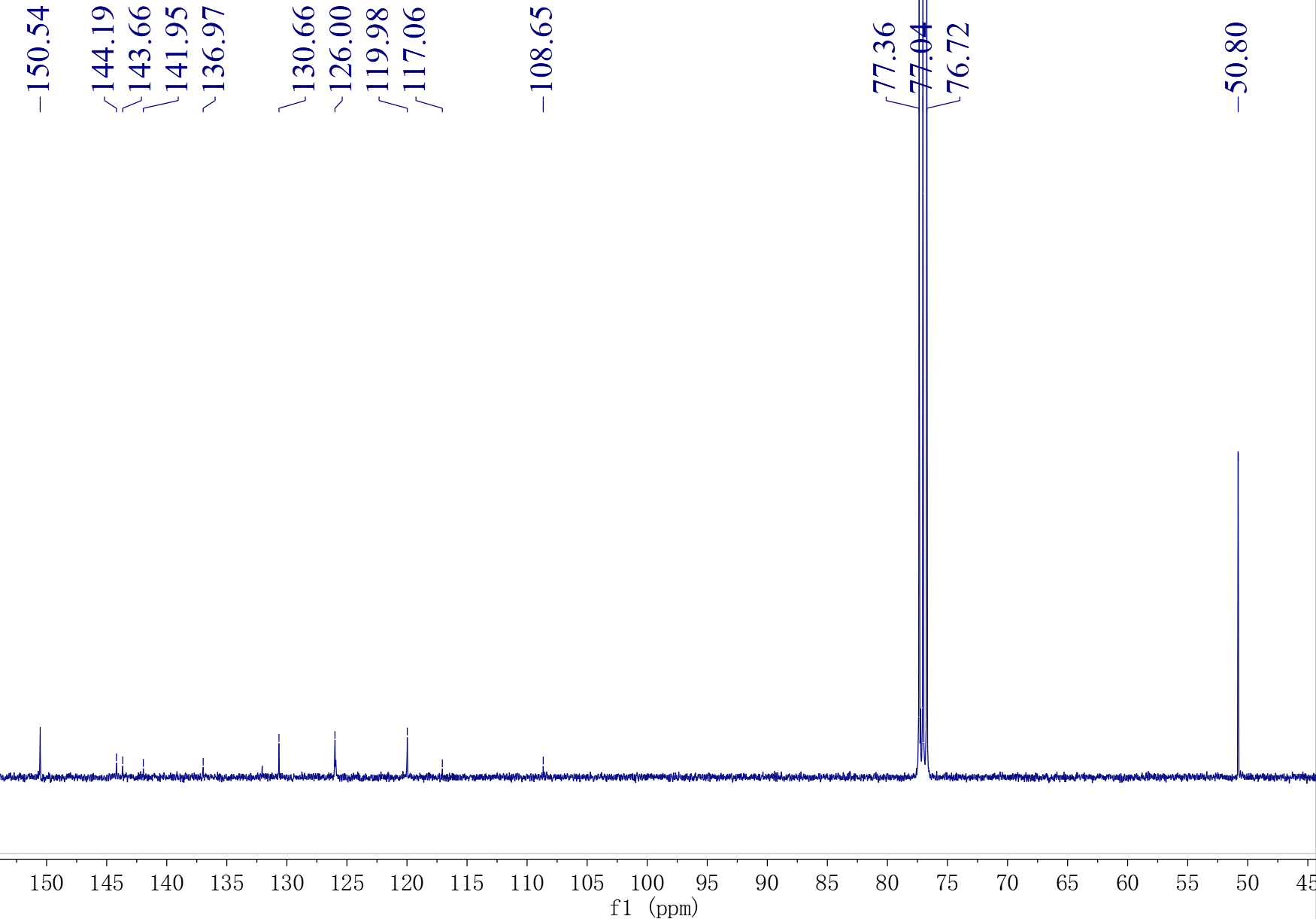


Figure S2. ^13^C NMR (400 MHz, 298K, CDCl_3_) spectrum of **TPPA**.


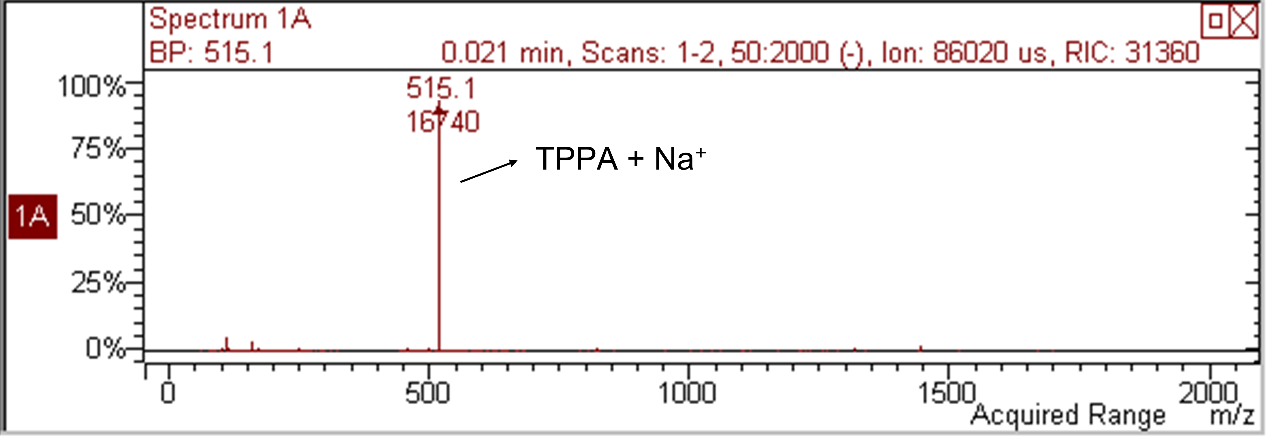


Figure S3. MS of **TPPA**. Calcd. for C_32_H_20_N_4_SNa ([M + Na]^+^): 515.1, found: 515.1.

1. Synthesis of **P5**^S2^

Scheme S2. Synthesis of pillar[5]arene (**P5**).

Pillar[5]arene was prepared according previous report, in a typical process, 1,4-diethoxybenzene (1.66 g, 1.0 mmol) and paraformaldehyde (0.30 g) were added to 60 mL ClCH_2_CH_2_Cl under vigorous stirring at room temperature. 1 mL BF_3_(Et_2_O) was added to the mixture and then reacted for 1 h. 50 mL H_2_O was added to stop the reaction, and pillar[5]arene was obtained by column chromatography (volume ratio: dichloromethane : petroleum ether = 1 : 1). White solid, 80%.

Figure S4. ^1^HNMR (400 MHz, 298K, CDCl_3_) spectrum of **P5**.

1. Impact of solvents
   1. In different organic solvents


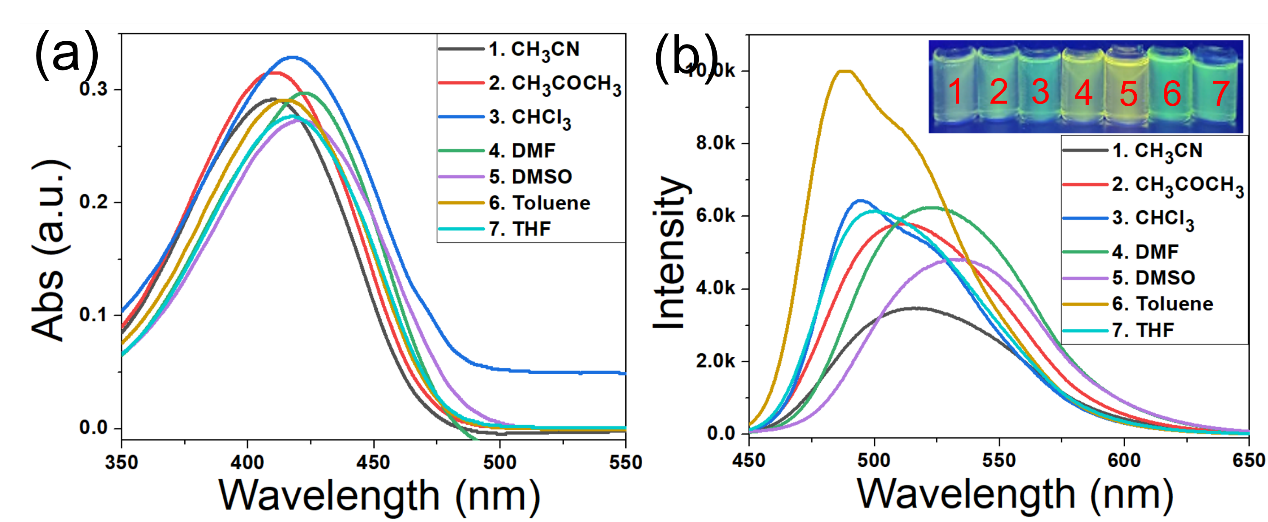


Figure S5. (a) absorption and (b) emission spectra of **TPPA** (0.4 mM, 4 mL) in different solvents (10 mL).

- 1. self-assembly in THF/H_2_O mixture

Table S1. Fluorescence quantum yield (FLQY) of TPPA dispersions in the binary solvent mixtures of water and THF with the composition variation. FLQY was estimated using quinine sulphate as the reference dye through triplicate measurements.

| System | Quantum yield |
| --- | --- |
| 10% water/90% THF | 9.46 ± 0.22 |
| 20% water/80% THF | 9.04 ± 0.23 |
| 30% water/70% THF | 8.07 ± 0.31 |
| 40% water/60% THF | 7.52 ± 0.21 |
| 50% water/50% THF | 6.83 ± 0.19 |
| 60% water/40% THF | 6.55 ± 0.32 |
| 70% water/30% THF | 5.21 ± 0.12 |
| 80% water/20% THF | 4.62 ± 0.11 |
| 90% water/10% THF | 2.53 ± 0.05 |

4.3 Self-assembly in DMSO/H_2_O mixture


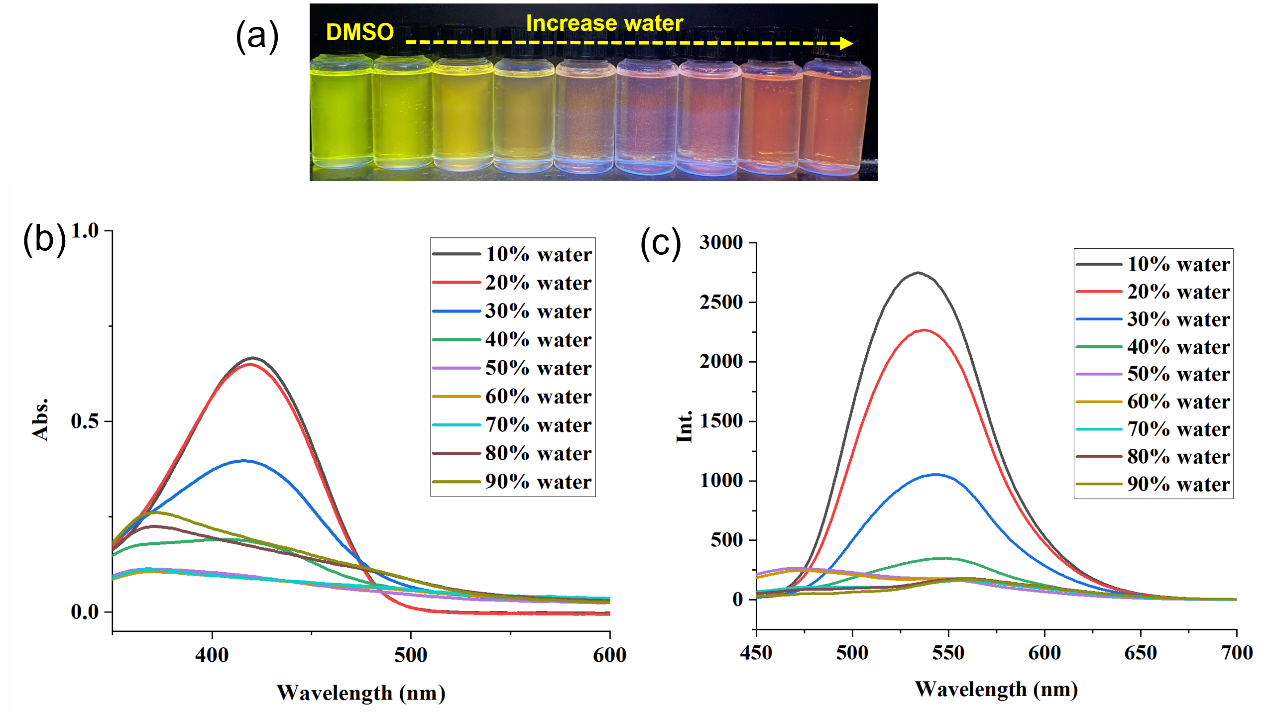


Figure S6. (a) absorption and (b) emission spectra of **TPPA** (0.4 mM, 4 mL) in 10 mL DMSO/water mixture with varying the water fraction from 0 to 90% with 10% of interval.

Table S2. Fluorescence quantum yield (FLQY) of TPPA dispersions in the binary solvent mixtures of water and DMSO with the composition variation. FLQY was estimated using quinine sulphate as the reference dye through triplicate measurements.

| System | Quantum yield |
| --- | --- |
| 10% water/90% DMSO | 9.55 ± 0.22 |
| 20% water/80% DMSO | 8.72 ± 0.23 |
| 30% water/70% DMSO | 5.34 ± 0.15 |
| 40% water/60% DMSO | 2.21 ± 0.13 |
| 50% water/50% DMSO | 2.02 ± 0.19 |
| 60% water/40% DMSO | 1.92 ± 0.11 |
| 70% water/30% DMSO | 1.85 ± 0.09 |
| 80% water/20% DMSO | 1.75 ± 0.08 |
| 90% water/10% DMSO | 1.73 ± 0.05 |

1. Cell viability


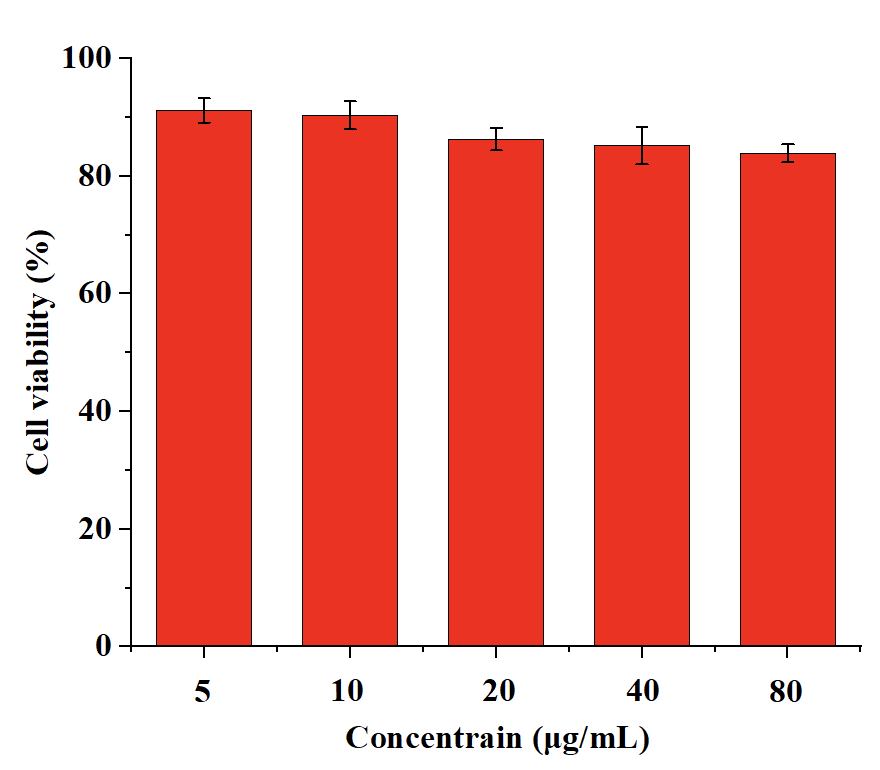


Figure S7. Cell viabilities of HeLa cells cultivated with **TPPA**.

1. White-light emission


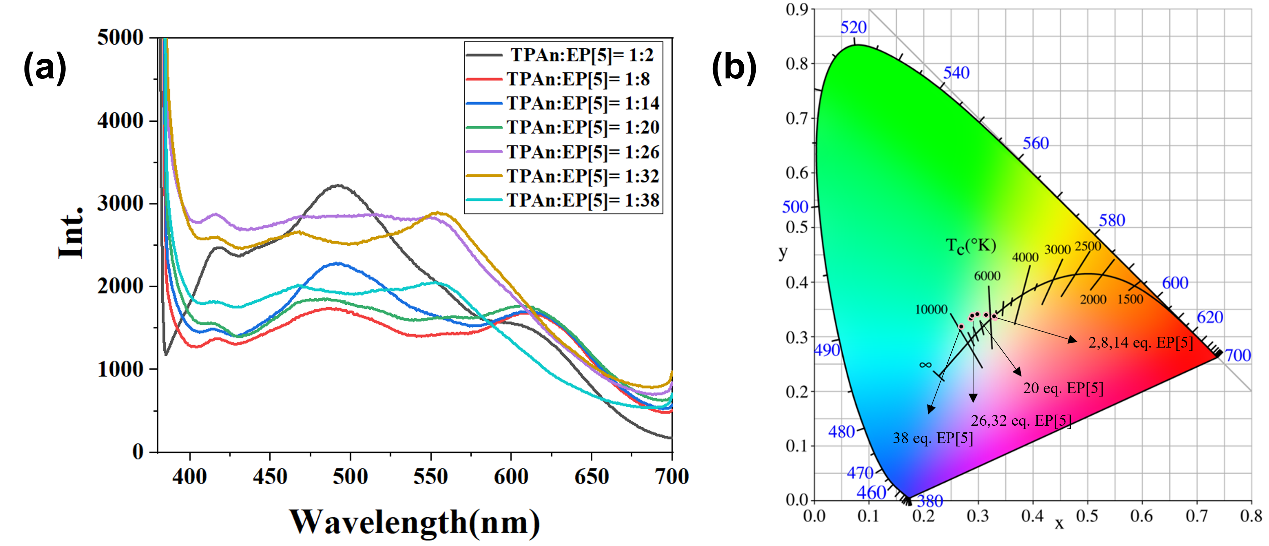


Figure S8. (a)The emission($\lambda ex$=365nm) spectra of TPPA (0.1 μM) and different proportions of **P5** in 95% water/5% THF. (b) CIE 1931 diagram.

References

S1. Kundu, S., Chowdhury, A., Nandi, S., Bhattacharyya, K., Patra, A. (2021) Deciphering the evolution of supramolecular nanofibers in solution and solid-state: a combined microscopic and spectroscopic approach. *Chem. Sci.* 12, 5874-5882. Doi: 10.1039/d0sc07050e.

S2. Zhang, R., Wang, C., Long, R., Chen, T., Yan, C. and Yao, Y. (2019) Pillar[5]arene Based [1]rotaxane Systems With Redox-Responsive Host-Guest Property: Design, Synthesis and the Key Role of Chain Length. *Front. Chem.* 7, 508. Doi: 10.3389/fchem.2019.00508.


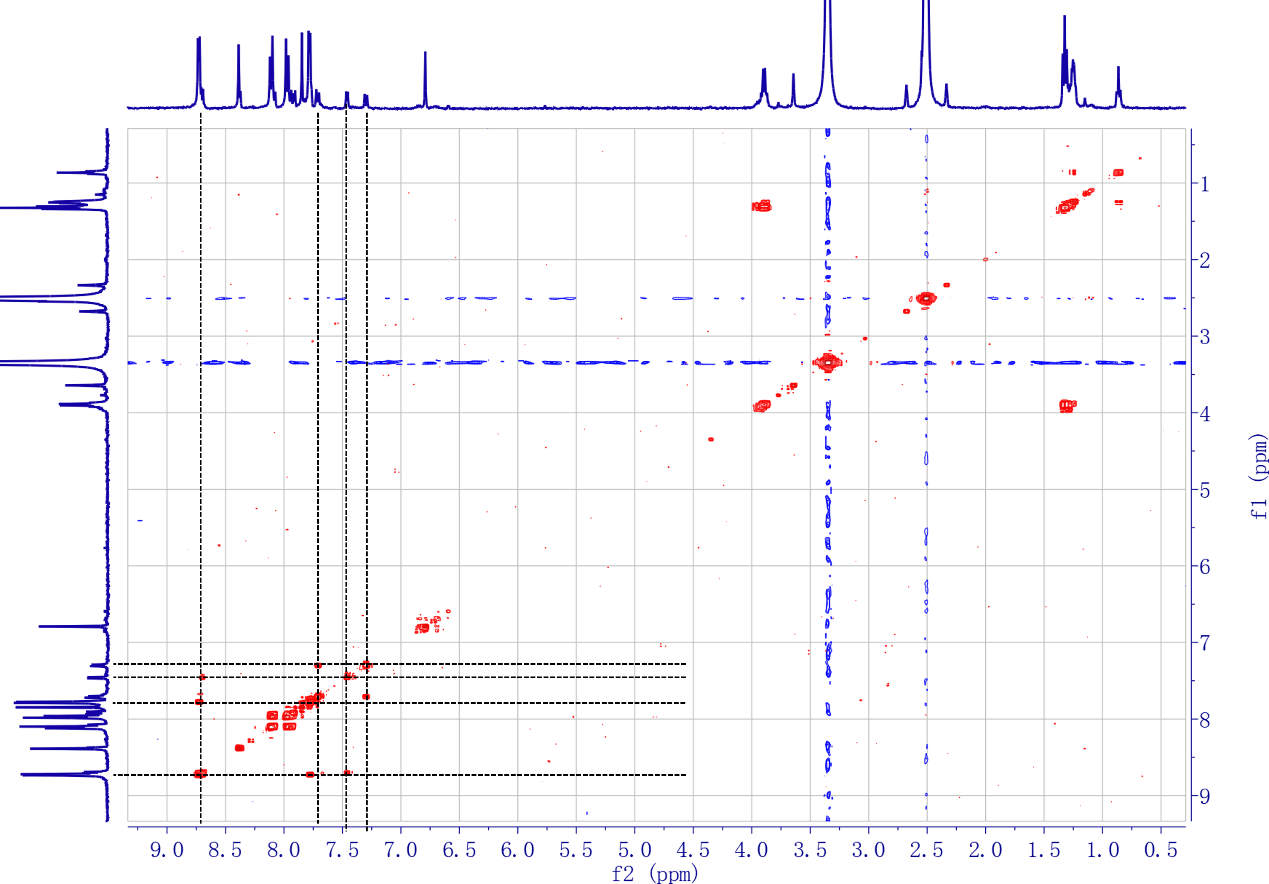


Figure S9. 2D NMR spectrum of TPPA@P5.


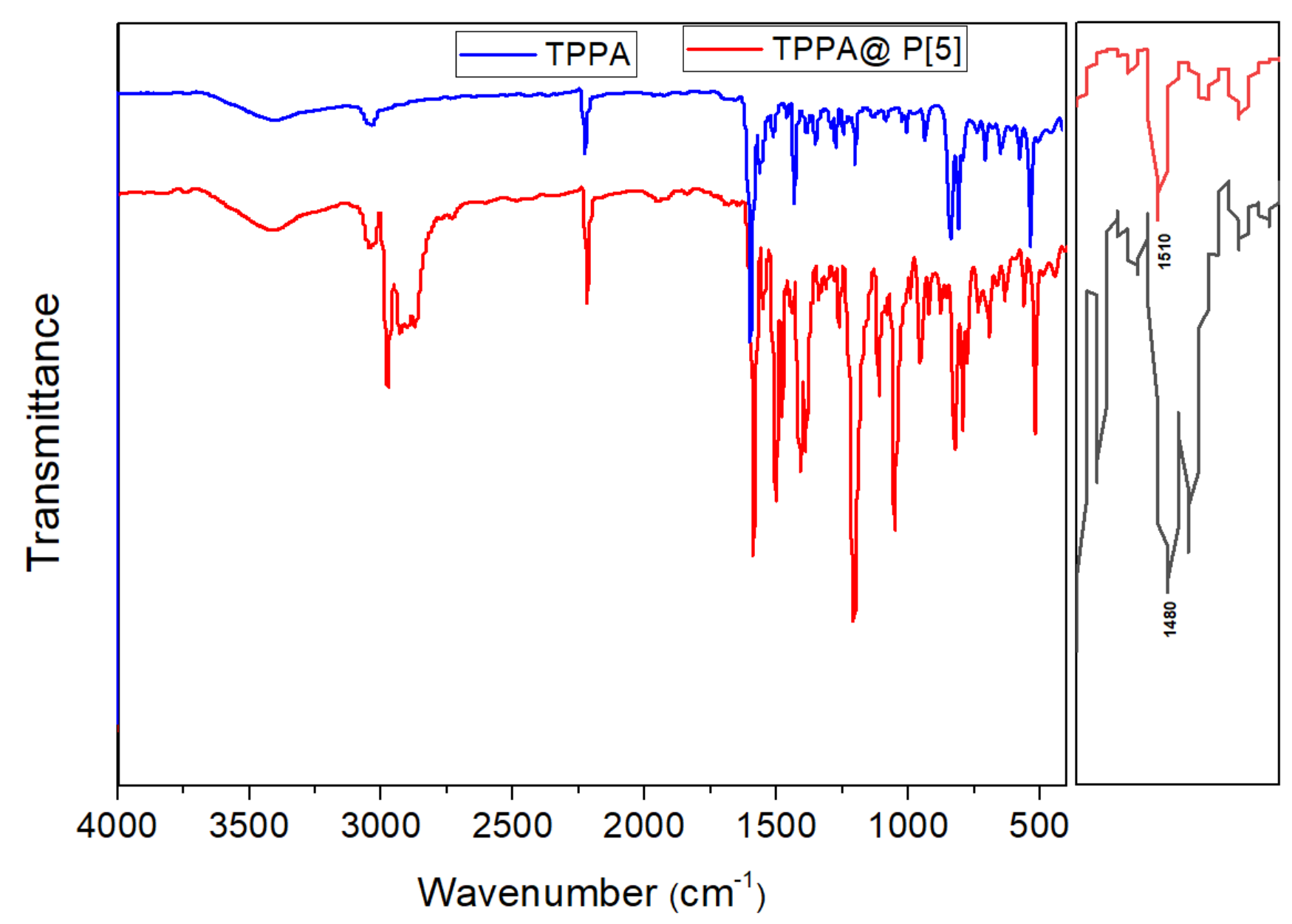


Figure S10. IR spectrum of TPPA@P5.


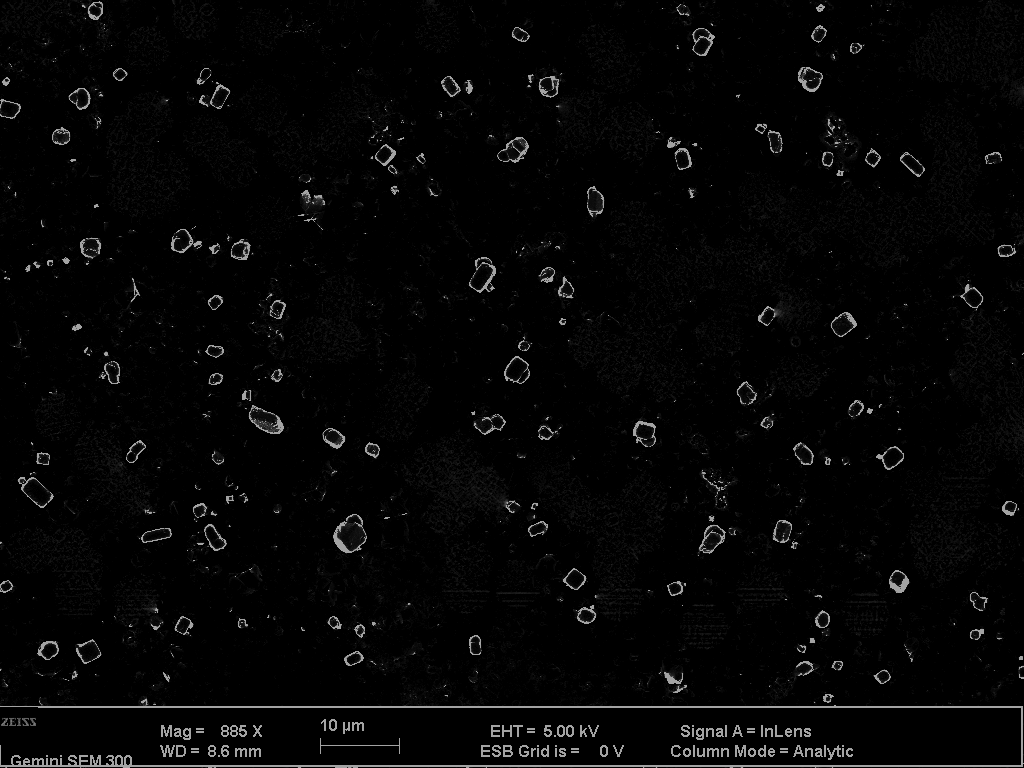


Figure S11 SEM image of TPPA@P5. 0.1 μmol TPPA and 2.0 μmol P5 in 5:95 of THF/H_2_O mixture.


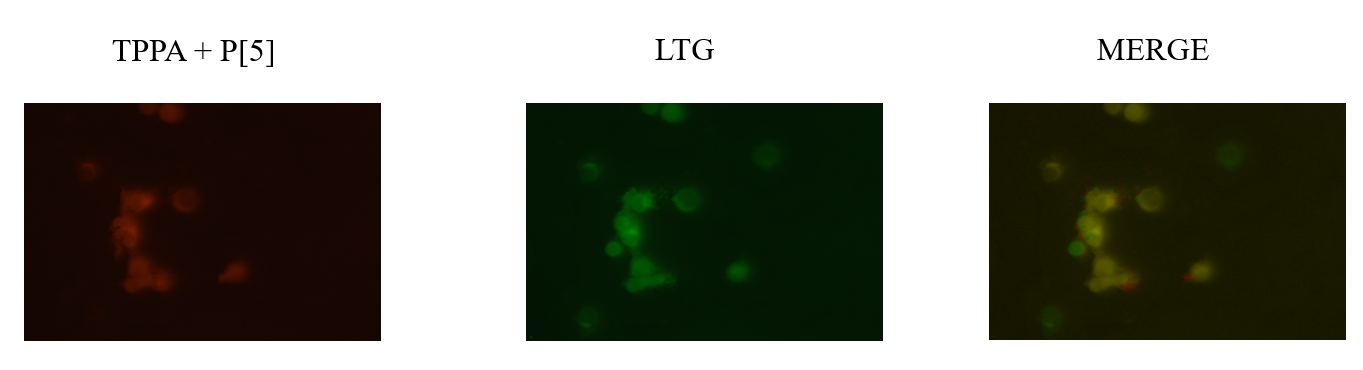


Figure S12 Confocal images of live HepG2 cells after incubation with 0.1 μmol TPPA and 2.0 μmol P5 in 5:95 of THF/H_2_O mixture (5.00 × 10^−4^ M) for 4 h.
